# Prolonged Glucocorticoid Exposure Does Not Accelerate Telomere Shortening in Cultured Human Fibroblasts

**DOI:** 10.3390/genes11121425

**Published:** 2020-11-27

**Authors:** Anthony S. Zannas, Oksana Kosyk, Calvin S. Leung

**Affiliations:** 1 Department of Psychiatry, University of North Carolina, Chapel Hill, NC 27599, USA; oksana_kosyk@med.unc.edu (O.K.); calvin_leung@med.unc.edu (C.L.); 2 Department of Genetics, University of North Carolina, Chapel Hill, NC 27514, USA; 3 Department of Psychiatry and Behavioral Sciences, Duke University Medical Center, Durham, NC 27710, USA; 4 Carolina Stress Initiative, University of North Carolina School of Medicine, Chapel Hill, NC 27514, USA

**Keywords:** cortisol, dexamethasone, *FKBP5*, glucocorticoid, stress, telomere

## Abstract

Psychosocial stress, especially when chronic or excessive, can increase disease risk and accelerate biological aging. Although the underlying mechanisms are unclear, in vivo studies have associated exposure to stress and glucocorticoid stress hormones with shorter telomere length. However, the extent to which prolonged glucocorticoid exposure can shorten telomeres in controlled experimental settings remains unknown. Using a well-characterized cell line of human fibroblasts that undergo gradual telomere shortening during serial passaging in culture, we show that prolonged exposure (up to 51 days) to either naturalistic levels of the human endogenous glucocorticoid cortisol or the more potent synthetic glucocorticoid dexamethasone is not sufficient to accelerate telomere shortening. While our findings await extension in other cell types and biological contexts, they indicate that the in vivo association of psychosocial stress with telomere shortening is unlikely to be mediated by a direct and universal glucocorticoid effect on telomere length.

## 1. Introduction

Psychosocial stress is ubiquitous in modern societies and, especially when chronic or excessive, can increase disease risk and accelerate biological aging [1]. While the underlying mechanisms are unclear, studies in humans have linked exposure to diverse types of stress with shortening of telomeres, the repetitive nucleotide sequences that protect chromosome ends and become shorter with cell division and organismal aging [2,3,4]. Moreover, repetitive stressors occurring during the human lifespan have been found to synergistically influence telomere length [5], suggesting that prolonged stress exposure can exert cumulative effects on telomere homeostasis. 

Stressors are characterized by vast heterogeneity, yet they share an ability to trigger a conserved neuroendocrine response that culminates in systemic secretion of glucocorticoid stress hormones, primarily cortisol in humans [6]. Systemic glucocorticoids, in turn, can markedly influence the function of essentially every cell in the human body [7]. Interestingly, in vivo glucocorticoid exposure has been associated with telomere shortening in blood cells [8,9], though lack of association has also been reported [10]. However, given the heterogeneity and fluctuation of blood cell populations, it is unclear to what extent these associations represent glucocorticoid-driven effects on telomeres of specific susceptible cell types or changes in the proportion of blood cells, which have varying telomere lengths [11]. Moreover, relatively short exposure to high concentrations of synthetic glucocorticoids has been linked with decreased expression and activity of telomerase, the enzyme that maintains telomere length in some cells [12], and with telomere shortening in some cell types but not others [13,14]. However, to our knowledge, no experimental studies have examined the extent to which prolonged exposure to naturalistic glucocorticoid levels drives telomere shortening.

To address this knowledge gap, we used a well-characterized cell line of human fibroblasts. Fibroblasts are a suitable cell model in this context, given their finite replicative potential, gradual telomere shortening during serial passaging in culture [15], and demonstrated ability to respond to the molecular actions of glucocorticoids [16,17]. We hypothesized that, by mimicking the effects of chronic stress in culture, prolonged glucocorticoid exposure would accelerate telomere shortening during cell passaging.

## 2. Materials and Methods 

### 2.1. Cell Culture and Treatments

Human IMR-90 (fetal lung fibroblast) cells were obtained from the Coriell Institute Cell Repository and were maintained in no-phenol-red Dulbecco’s Modified Eagle Medium (DMEM) supplemented with 15% fetal bovine serum, high glucose, sodium pyruvate, l-glutamine, non-essential amino acids, and antibiotic/antimycotic. Cells were grown in a humidified incubator at 37 °C and 5% CO_2_. All cultures were seeded at a cell density of 10,000 cells/cm^2^ and allowed to proliferate for four to five days or until the cells reached 90% confluency. Cortisol and dexamethasone were continuously added to cultures for the indicated duration of time, each at a final concentration of 100 nM. Both compounds were diluted in a very low final amount of DMSO (0.0001%), and the same DMSO concentration was used as vehicle control. All compounds were purchased from Sigma-Aldrich (St. Louis, MO, USA) and were replaced in fresh media every two to three days throughout treatment.

### 2.2. DNA and RNA Extraction

IMR-90 cells were collected while still replicating at 0, 24, and 51 days after continuous treatment with DMSO, cortisol, or dexamethasone as indicated. DNA was extracted using the Genfind V3 DNA extraction kit (Beckman Coulter, Brea, CA, USA) according to manufacturer instructions. DNA concentration and purity were determined using the Take3 microplate spectrophotometer (BioTek Instruments, Winooski, VT, USA). Total RNA was isolated using the RNeasy Mini Kit (QIAGEN, Germantown, MD, USA). In total, 200 ng of total RNA was then treated with ezDNase (ThermoFisher, Waltham, MA, USA) to degrade carry-over genomic DNA. cDNA was synthesized using the SuperScript IV VILO Master Mix (ThermoFisher, Waltham, MA, USA). A total of 4 ng of cDNA from each sample was added to wells across an optical 96-well reaction plate (Applied Biosystems, Foster City, CA, USA) for qPCR measurement.

### 2.3. qPCR for Telomere Length and Glucocorticoid-Responsive Gene Expression

All qPCR experiments were performed using the QuantStudio 6 Flex Real-Time PCR System (Applied Biosystems, Foster City, CA, USA) under standard cycling conditions. 

To confirm that prolonged glucocorticoid exposure influences gene expression in IMR-90 cells, we measured mRNA levels of the established glucocorticoid-responsive gene *FKBP5* [18]. *FKBP5* and *GAPDH* (housekeeping gene) primers were diluted to a final concentration of 300 nM in the two separate master mixes using the PowerUp SYBR Green Master Mix (Applied Biosystems, Foster City, CA, USA). *FKBP5* and *GAPDH* PCRs were performed in the same 96-well reaction plate. Fold change was calculated using the formula: 2^−(ΔCt^_treatment_^−ΔCt^_vehicle_^)^ = 2^−ΔΔCt^. The primer sequences were as follows: *FKBP5* F: 5′-CAAAAGAGTGGGGAATGGTG-3′; *FKBP5* R: 5′-CCTTGATGACTTGGCCTTTG-3′; *GAPDH* F: 5′-TCACCAGGGCTGCTTTTAAC-3′; *GAPDH* R: 5′-ATGACAAGCTTCCCGTTCTC-3′. 

Relative telomere length was measured using an established qPCR-based method that determines the Telomere/Single Copy Gene (SCG) ratio or T/S ratio [19]. Samples were run as technical duplicates when possible. In total, 100 ng of genomic DNA from each sample was added to wells across an optical 96-well reaction plate. Primers were diluted to a final concentration of 300 nM in the two separate master mixes using the PowerUp SYBR Green Master Mix (Applied Biosystems, Foster City, CA, USA). Telomere and SCG PCRs were performed in the same 96-well reaction plate. The T/S ratio was calculated using the following formula: [2^Ct(telomere)^/2^Ct(SCG)^] ^−1^ = 2^−ΔCt^. The relative T/S ratio (comparing different treatments) is: 2^− (ΔCt^_1_^−ΔCt^_2_^)^ = 2^−ΔΔCt^. Standard primers for telomere hexamer repeats and *β*-Globin (the SCG used in this method) were previously described [20]: Telo1: 5′-CGGTTTGTTTGGGTTTGGGTTTGGGTTTGGGTTTGGGTT-3′; Telo2: 5′-GGCTTGCCTTACCCTTACCCTTACCCTTACCCTTACCCT-3′; SCG1: 5′-GCTTCTGACACAACTGTGTTCACTAGC-3′; SCG2: 5′-CACCAACTTCATCCACGTTCACC-3′.

### 2.4. Statistical Analysis

To examine the effect of glucocorticoid (cortisol or dexamethasone) exposure and culture duration on telomere length, we performed two-way analysis of variance (ANOVA) with treatment (vehicle vs. cortisol, or vehicle vs. dexamethasone) and cell passage (early, middle, late) as the two factors of interest. Both main effects and interactions were examined. The effect of glucocorticoid (cortisol or dexamethasone) on *FKBP5* expression was determined using Student’s *t*-test for each cell passage (middle, late). All p-values were 2-tailed, and the level of statistical significance was set a priori at *α* = 0.05. All statistical analyses were conducted with R version 3.5.1. 

## 3. Results

IMR-90 cells underwent prolonged exposure to either vehicle or glucocorticoid (as indicated), and telomere length was measured at three time points (cell passages): 0 days (“early”), to determine the baseline telomere length right before treatment onset; 24 days (“middle”), an intermediate time point suitable for assessing potential cumulative effects of treatment on telomere length; 51 days (“late”), the latest time point following treatment completion. To determine the extent to which naturalistic stress hormone exposure accelerates telomere shortening, we used the primary endogenous human glucocorticoid cortisol at a concentration of 100 nM, which can be reached in human tissues during in vivo stress [21,22,23]. To further address the potential role of glucocorticoid type and potency, cells were also separately treated with the considerably more potent and commonly used synthetic glucocorticoid dexamethasone [24] at the same concentration (100 nM). 

**Figure 1:**
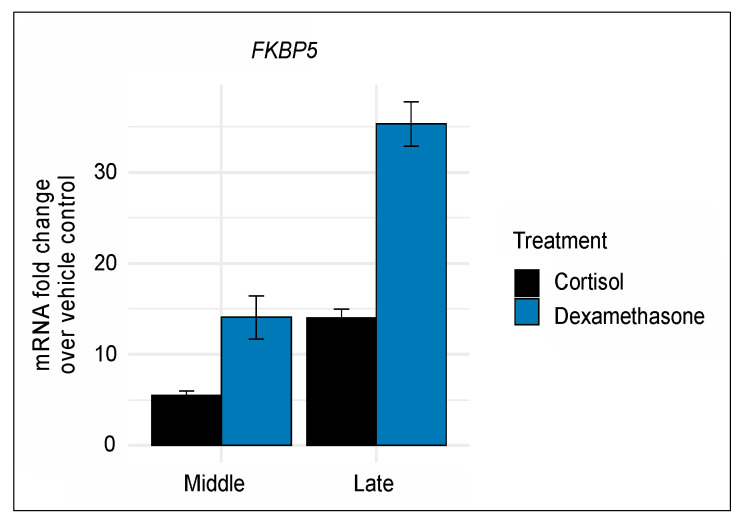
Prolonged glucocorticoid exposure to 100 nM of either cortisol or dexamethasone induces robust and cumulative gene induction as indicated by mRNA levels of the known glucocorticoid-responsive gene *FKBP5*. Each time point included 6 biological replicates for the vehicle and cortisol group and 3 biological replicates for the dexamethasone group. For each time point, mRNA levels were normalized and are shown as fold change compared to vehicle control. Error bars represent one standard error above and below the mean.

As a first step, we sought to confirm that prolonged exposure to either compound exerts significant molecular actions in our cell model. Shorter treatment duration with 100 nM of either cortisol or glucocorticoid was previously shown to induce gene transcription in IMR-90 cells [16,17]. Furthermore, we confirmed that prolonged exposure to 100 nM of either compound robustly induced gene transcription as indicated by mRNA levels of the known glucocorticoid-responsive gene *FKBP5* [18] (Figure 1). Notably, the glucocorticoid-driven induction of *FKBP5* accrued across middle and late passages and, as expected, was more pronounced for the more potent dexamethasone (middle fold change = 14.1, t_7_ = 8.4; late fold change = 35.3, t_7_ = 21.5; both *p*-values < 0.0001) than for cortisol (middle fold change = 5.5, t_10_ = 9.9; late fold change = 14.0, t_10_ = 13.5; both *p*-values < 0.0001).

**Figure 2:**
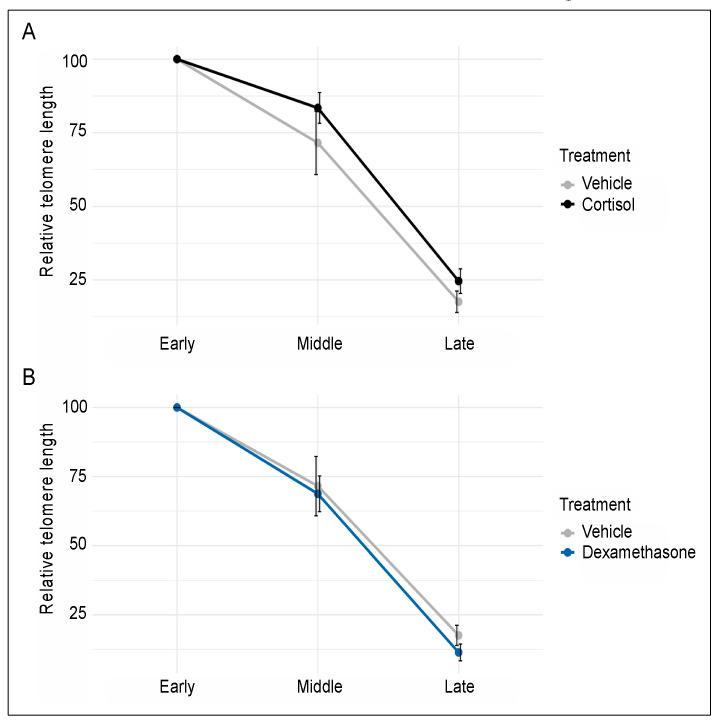
Prolonged exposure to 100 nM of either cortisol (**A**) or dexamethasone (**B**) does not influence the rate of telomere shortening observed during passaging of human fibroblasts. Telomere length was determined at early, middle, and late passage (0, 24, and 51 days of treatment, respectively). Each time point included 6 biological replicates for the vehicle and cortisol group and 5 biological replicates for the dexamethasone group. Relative telomere lengths were normalized and are shown as percent of the early passage cells. Error bars represent one standard error above and below the mean.

Although cell passaging (time in culture) resulted in telomere shortening (as expected), none of the two glucocorticoids significantly influenced telomere length. More specifically, two-way ANOVA of cells treated in parallel with either vehicle or cortisol showed a significant main effect of time (F_2,25_ = 64.23, *p* < 0.0001), but cortisol did not affect telomere length through either a main effect (F_1,25_ = 2.17, *p* = 0.15) or an interaction with time (F_1,25_ = 0.14, *p* = 0.71) (Figure 2A). Similarly, two-way ANOVA of cells treated in parallel with either vehicle or dexamethasone showed a significant main effect of time (F_2,23_ = 58.09, *p* < 0.0001), but dexamethasone did not influence telomere length through either a main effect (F_1,23_ = 0.43, *p* = 0.52) or an interaction with time (F_1,23_ = 0.06, *p* = 0.81) (Figure 2B). Together, these findings indicate that prolonged exposure to biologically meaningful concentrations of either cortisol or dexamethasone is not sufficient to accelerate telomere shortening in cultured human fibroblasts.

## 4. Discussion

In vivo studies have associated telomere shortening with psychosocial stress and glucocorticoid burden [2,3,4,8,9], but the molecular mechanisms underlying these associations remain unknown. In the present study, we examined the extent to which prolonged exposure to either naturalistic stress levels of the endogenous human glucocorticoid cortisol or the more potent synthetic glucocorticoid dexamethasone may accelerate telomere shortening in cultured human fibroblasts, but we found no evidence for such an effect. 

Although our findings await extension to other cell types and biological contexts, the lack of telomere shortening with either cortisol or dexamethasone exposure in our established cellular model indicates that the observed in vivo associations may be explained by more complex or alternative mechanisms. In addition to cortisol, stress triggers a multitude of molecular effectors and changes in living humans; for example, higher levels of perceived stress have been associated with elevation in markers of oxidative DNA damage [25], a process known to contribute to telomere attrition in multiple cell types including fibroblasts [26,27,28]. Stress and glucocorticoids have been further posited to shorten telomeres through metabolic perturbations at multiple organismal levels [9,29]. Moreover, the majority of human studies to date have measured telomeres in mixtures of heterogeneous blood cells, thus allowing for two additional, non-mutually exclusive interpretations. First, it is possible that stress and glucocorticoids affect only selected, susceptible cell types. In line with this possibility, shorter exposure to higher dexamethasone concentrations has been shown to decrease telomerase expression and activity and to shorten telomeres in some epithelial and cancer cell types but not others [13,14]. Alternatively, the observed in vivo associations may be explained, in part, by stress-related changes in the proportion of blood cells, which are known to have varying telomere lengths [11]. The latter possibility may be addressed by studies that adjust for blood cell proportions or, preferably, measure telomere length in purified cell types. Such convergent mechanistic work in cultured cells and living organisms may ultimately enhance our ability to prevent the potentially deleterious effects of stress exposure on telomere homeostasis.

While these intriguing questions remain to be addressed by future studies, the present report builds upon a body of evidence associating psychosocial stress with telomere attrition, indicating that this association is unlikely to be mediated by a direct and universal effect of prolonged glucocorticoid exposure on telomere length.
